# *N*-(5-Nitro-1,3-thia­zol-2-yl)-4-(tri­fluoro­meth­yl)benzamide

**DOI:** 10.1107/S1600536813011264

**Published:** 2013-05-22

**Authors:** Xi-Wang Liu, Han Zhang, Ya-Jun Yang, Jian-Yong Li, Ji-Yu Zhang

**Affiliations:** a Key Laboratory of New Animal Drug Project, Gansu Province, Key Laboratory of Veterinary Pharmaceutical Development, Ministry of Agriculture, Lanzhou Institute of Animal Science and Veterinary Pharmaceutics of CAAS, Lanzhou 730050, People’s Republic of China

## Abstract

There are two independent and conformationally dissimilar mol­ecules (*A* and *B*) in the asymmetric unit of the title compound, C_11_H_6_F_3_N_3_O_3_S; the dihedral angles between the benzene and thia­zole rings are 33.8 (2)° in *A* and 59.7 (2)° in *B*. The similarity of the C—N bond lengths in the amide group [1.379 (5) and 1.358 (5) Å for *A*, and 1.365 (5) and 1.363 (5) Å for *B*] indicates the presence of conjugation between the two rings. In the crystal, mol­ecules are linked by N—H⋯N hydrogen bonds, forming chains extending along [010]; weak N—H⋯O_amide_ inter­actions are also present in the structure.

## Related literature

For the anti­parasitic activity of nitazoxanide, see: Fox & Saravolatz (2005 ▶) and for the anti­bacterial activity of thia­zolides, see: Gargala *et al.* (2010 ▶); Stachulski *et al.* (2011 ▶). For the synthesis and anti­bacterial activity of the title compound, see: Ballard *et al.* (2011 ▶).
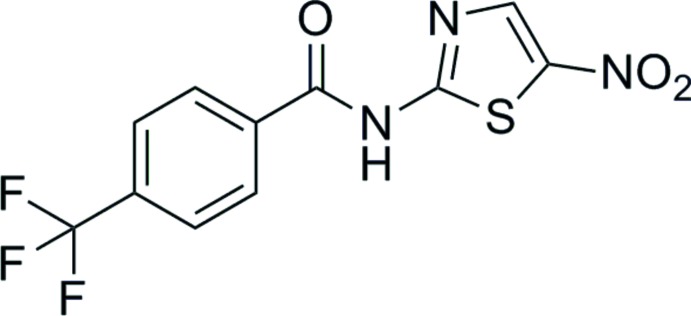


## Experimental

### 

#### Crystal data

C_11_H_6_F_3_N_3_O_3_S
*M* *_r_* = 317.26
Monoclinic, 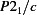

*a* = 12.362 (4) Å
*b* = 8.946 (3) Å
*c* = 23.261 (8) Å
β = 100.762 (4)°
*V* = 2527.2 (15) Å^3^
*Z* = 8
Mo *K*α radiation
μ = 0.31 mm^−1^
*T* = 296 K
0.34 × 0.32 × 0.25 mm

#### Data collection

Bruker APEXII CCD diffractometer
Absorption correction: multi-scan (*SADABS*; Sheldrick, 1996 ▶) *T*_min_ = 0.903, *T*_max_ = 0.927
11671 measured reflections
4588 independent reflections
3183 reflections with *I* > 2σ(*I*)
*R*_int_ = 0.039

#### Refinement

*R*[*F* ^2^ > 2σ(*F* ^2^)] = 0.069
*wR*(*F* ^2^) = 0.175
*S* = 1.06
4588 reflections
379 parameters
H-atom parameters constrained
Δρ_max_ = 0.50 e Å^−3^
Δρ_min_ = −0.48 e Å^−3^

### 

Data collection: *APEX2* (Bruker, 2008 ▶); cell refinement: *SAINT* (Bruker, 2008 ▶); data reduction: *SAINT*; program(s) used to solve structure: *SHELXS97* (Sheldrick, 2008 ▶); program(s) used to refine structure: *SHELXL97* (Sheldrick, 2008 ▶); molecular graphics: *SHELXTL* (Sheldrick, 2008 ▶); software used to prepare material for publication: *SHELXTL*.

## Supplementary Material

Crystal structure: contains datablock(s) I, global. DOI: 10.1107/S1600536813011264/zs2257sup1.cif

Structure factors: contains datablock(s) I. DOI: 10.1107/S1600536813011264/zs2257Isup2.hkl

Supplementary material file. DOI: 10.1107/S1600536813011264/zs2257Isup3.cml

Additional supplementary materials:  crystallographic information; 3D view; checkCIF report

## Figures and Tables

**Table 1:** Hydrogen-bond geometry (Å, °)

*D*—H⋯*A*	*D*—H	H⋯*A*	*D*⋯*A*	*D*—H⋯*A*
N1—H1⋯N5^i^	0.86	2.32	3.044 (5)	142
N1—H1⋯O3^i^	0.86	2.57	3.023 (5)	115
N4—H4⋯N2^ii^	0.86	2.18	2.939 (5)	147
N4—H4⋯O2	0.86	2.62	3.146 (5)	121

Symmetry codes: (i) 
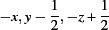
; (ii) 
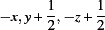
.

**Fig. 1.**
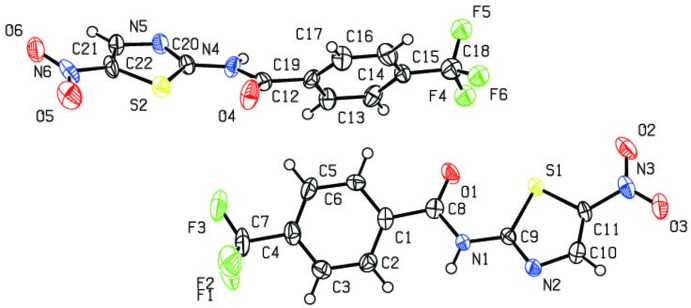
The molecular conformation and atom numbering scheme for the two independent molecules in the asymmetric unit of title compound, with displacement ellipsoids drawn at the 30% probability level.

## supplementary crystallographic information

### Comment

Parasitic and bacterial infections represnt a significant cause of morbidity and mortality worldwide (Fox & Saravolatz, 2005). Nitazoxanide is a novel antiparasitic compound developed for both human and animal use and is the parent compound of a class of drugs named thiazolides. Recently, a number of thiazolides were synthesized and their biological activities were also evaluated (Gargala *et al*., 2010); Stachulski *et al.*, 2011). The title compound C_11_H_6_F_3_N_3_O_3_S is a new thiazolide which displays higher antibacterial activity than nitazoxanide (Ballard *et al.*, 2011) and the crystal structure is reported herein.

In this structure, there are two independent and conformationally dissimilar molecules in the asymmetric unit (Fig. 1) [dihedral angles between the benzene and thiazole rings: 33.8 (2) and 59.7 (2)°]. In the crystal, the molecules are linked by intermolecular N—H···N hydrogen bonds (Table 2), giving one-dimensional chains extending along [010]. Also present in the structure are intra- and intermolecular N—H···O_amide_ interactions within the chains.

The similarity of the C—N bond lengths [N1—C8, 1.379 (5) Å and N1—C9, 1.358 (5) Å] in the amide group indicates the presence of conjugation between the two rings, which confirms the hypothetical mechanism with thiazolides that the amin anion may interact with the target PFOR enzyme.

### Experimental

To a solution of 5-nitrothiazol-2-amine (1 mmol) in distilled pyridine was added a equimolar amount of 4-trifluorobenzoyl chloride with stirring. After the addition was complete, the reaction mixture was allowed to stand overnight. The reaction was judged complete by TLC analysis. The crude product that separated on dilution was filtered, washed with 10% sodium bicarbonate solution, then several times with water. The dry solid was purified by chromatography to give the pure title compound and crystals suitable for single crystal X-ray analysis were obtained by recrystallization from methanol.

### Refinement

The positions of all H atoms were determined geometrically and refined using a riding model with C—H = 0.93 Å and N—H = 0.86 Å and with *U*_iso_(H) = 1.2*U*_eq_(C, N).

### Crystal data

**Table:**

C_11_H_6_F_3_N_3_O_3_S	*F*(000) = 1280
*M**_r_* = 317.26	*D*_x_ = 1.668 Mg m^−^^3^
Monoclinic, *P*2_1_/*c*	Melting point = 487–489 K
Hall symbol: -P 2ybc	Mo *K*α radiation, λ = 0.71073 Å
*a* = 12.362 (4) Å	Cell parameters from 2723 reflections
*b* = 8.946 (3) Å	θ = 2.4–24.6°
*c* = 23.261 (8) Å	µ = 0.31 mm^−^^1^
β = 100.762 (4)°	*T* = 296 K
*V* = 2527.2 (15) Å^3^	Block, colourless
*Z* = 8	0.34 × 0.32 × 0.25 mm

### Data collection

**Table:**

Bruker APEXII CCD diffractometer	4588 independent reflections
Radiation source: fine-focus sealed tube	3183 reflections with *I* > 2σ(*I*)
Graphite monochromator	*R*_int_ = 0.039
φ and ω scans	θ_max_ = 25.5°, θ_min_ = 2.2°
Absorption correction: multi-scan (*SADABS*; Sheldrick, 1996)	*h* = −14→14
*T*_min_ = 0.903, *T*_max_ = 0.927	*k* = −9→10
11671 measured reflections	*l* = −28→26

### Refinement

**Table:**

Refinement on *F*^2^	Primary atom site location: structure-invariant direct methods
Least-squares matrix: full	Secondary atom site location: difference Fourier map
*R*[*F*^2^ > 2σ(*F*^2^)] = 0.069	Hydrogen site location: inferred from neighbouring sites
*wR*(*F*^2^) = 0.175	H-atom parameters constrained
*S* = 1.06	*w* = 1/[σ^2^(*F*_o_^2^) + (0.0692*P*)^2^ + 3.2322*P*] where *P* = (*F*_o_^2^ + 2*F*_c_^2^)/3
4588 reflections	(Δ/σ)_max_ = 0.001
379 parameters	Δρ_max_ = 0.50 e Å^−^^3^
0 restraints	Δρ_min_ = −0.48 e Å^−^^3^

### Special details

**Table:**

Geometry. All e.s.d.'s (except the e.s.d. in the dihedral angle between two l.s. planes) are estimated using the full covariance matrix. The cell e.s.d.'s are taken into account individually in the estimation of e.s.d.'s in distances, angles and torsion angles; correlations between e.s.d.'s in cell parameters are only used when they are defined by crystal symmetry. An approximate (isotropic) treatment of cell e.s.d.'s is used for estimating e.s.d.'s involving l.s. planes.
Refinement. Refinement of *F*^2^ against ALL reflections. The weighted *R*-factor *wR* and goodness of fit *S* are based on *F*^2^, conventional *R*-factors *R* are based on *F*, with *F* set to zero for negative *F*^2^. The threshold expression of *F*^2^ > σ(*F*^2^) is used only for calculating *R*-factors(gt) *etc*. and is not relevant to the choice of reflections for refinement. *R*-factors based on *F*^2^ are statistically about twice as large as those based on *F*, and *R*- factors based on ALL data will be even larger.

### Fractional atomic coordinates and isotropic or equivalent isotropic displacement parameters (Å^2^)

**Table:**

	*x*	*y*	*z*	*U*_iso_*/*U*_eq_	
C1	0.2871 (3)	0.2306 (5)	0.10652 (16)	0.0387 (10)	
C2	0.2390 (3)	0.1247 (5)	0.06679 (17)	0.0448 (11)	
H2	0.1799	0.0687	0.0743	0.054*	
C3	0.2787 (4)	0.1018 (5)	0.01590 (18)	0.0483 (11)	
H3	0.2467	0.0296	−0.0107	0.058*	
C4	0.3654 (3)	0.1855 (5)	0.00447 (18)	0.0471 (11)	
C5	0.4138 (4)	0.2911 (6)	0.0439 (2)	0.0549 (13)	
H5	0.4721	0.3481	0.0360	0.066*	
C6	0.3758 (3)	0.3125 (5)	0.09514 (19)	0.0498 (12)	
H6	0.4098	0.3821	0.1223	0.060*	
C7	0.4053 (5)	0.1639 (8)	−0.0515 (2)	0.0687 (16)	
C8	0.2493 (3)	0.2599 (5)	0.16232 (17)	0.0391 (10)	
C9	0.0895 (3)	0.2764 (5)	0.20673 (16)	0.0357 (9)	
C10	−0.0463 (4)	0.3137 (5)	0.25281 (17)	0.0459 (11)	
H10	−0.1190	0.3152	0.2583	0.055*	
C11	0.0372 (3)	0.3595 (5)	0.29379 (16)	0.0388 (10)	
C12	0.3086 (3)	0.8380 (5)	0.34839 (17)	0.0401 (10)	
C13	0.3064 (4)	0.7008 (5)	0.32116 (18)	0.0461 (11)	
H13	0.2820	0.6166	0.3384	0.055*	
C14	0.3405 (4)	0.6892 (5)	0.26822 (19)	0.0490 (11)	
H14	0.3404	0.5965	0.2502	0.059*	
C15	0.3746 (3)	0.8129 (5)	0.24206 (18)	0.0436 (10)	
C16	0.3762 (4)	0.9502 (6)	0.2691 (2)	0.0554 (12)	
H16	0.3984	1.0348	0.2512	0.066*	
C17	0.3448 (4)	0.9620 (6)	0.32261 (19)	0.0545 (12)	
H17	0.3481	1.0541	0.3414	0.065*	
C18	0.4075 (4)	0.8034 (7)	0.1841 (2)	0.0587 (13)	
C19	0.2738 (3)	0.8557 (5)	0.40602 (17)	0.0426 (10)	
C20	0.1252 (3)	0.8112 (5)	0.45709 (16)	0.0377 (9)	
C21	0.0747 (4)	0.8551 (5)	0.54822 (17)	0.0417 (10)	
C22	−0.0063 (4)	0.7988 (5)	0.50768 (17)	0.0454 (11)	
H22	−0.0763	0.7793	0.5151	0.054*	
F1	0.4126 (4)	0.0182 (5)	−0.06394 (15)	0.1155 (15)	
F2	0.3376 (3)	0.2148 (5)	−0.09695 (13)	0.0960 (12)	
F3	0.5002 (3)	0.2196 (7)	−0.05158 (16)	0.158 (2)	
F4	0.4257 (4)	0.6685 (5)	0.16786 (16)	0.1311 (18)	
F5	0.3324 (4)	0.8501 (5)	0.14227 (14)	0.1252 (17)	
F6	0.4934 (4)	0.8772 (8)	0.18052 (17)	0.184 (3)	
N1	0.1382 (3)	0.2403 (4)	0.16086 (13)	0.0382 (8)	
H1	0.0982	0.2039	0.1298	0.046*	
N2	−0.0176 (3)	0.2646 (4)	0.20246 (14)	0.0445 (9)	
N3	0.0305 (3)	0.4175 (4)	0.34971 (15)	0.0446 (9)	
N4	0.1728 (3)	0.7979 (4)	0.40900 (13)	0.0416 (9)	
H4	0.1375	0.7507	0.3791	0.050*	
N5	0.0219 (3)	0.7725 (4)	0.45493 (14)	0.0437 (9)	
N6	0.0665 (4)	0.8983 (4)	0.60621 (15)	0.0503 (10)	
O1	0.3090 (2)	0.3016 (4)	0.20642 (12)	0.0560 (9)	
O2	0.1160 (3)	0.4596 (4)	0.38111 (13)	0.0638 (9)	
O3	−0.0597 (3)	0.4226 (4)	0.36369 (13)	0.0587 (9)	
O4	0.3288 (2)	0.9184 (4)	0.44772 (13)	0.0632 (10)	
O5	0.1513 (3)	0.9334 (4)	0.63961 (14)	0.0713 (11)	
O6	−0.0250 (3)	0.8991 (4)	0.61955 (13)	0.0647 (10)	
S1	0.16224 (9)	0.34362 (13)	0.27209 (4)	0.0429 (3)	
S2	0.19504 (9)	0.88052 (14)	0.52279 (4)	0.0469 (3)	

### Atomic displacement parameters (Å^2^)

**Table:**

	*U*^11^	*U*^22^	*U*^33^	*U*^12^	*U*^13^	*U*^23^
C1	0.037 (2)	0.045 (3)	0.035 (2)	0.0080 (19)	0.0076 (17)	0.0032 (19)
C2	0.046 (3)	0.046 (3)	0.045 (2)	−0.001 (2)	0.014 (2)	−0.002 (2)
C3	0.051 (3)	0.051 (3)	0.044 (2)	0.002 (2)	0.012 (2)	−0.006 (2)
C4	0.041 (2)	0.062 (3)	0.039 (2)	0.012 (2)	0.0087 (19)	0.004 (2)
C5	0.041 (3)	0.072 (4)	0.056 (3)	−0.003 (2)	0.020 (2)	0.000 (3)
C6	0.041 (2)	0.061 (3)	0.048 (3)	−0.008 (2)	0.010 (2)	−0.011 (2)
C7	0.063 (3)	0.103 (5)	0.046 (3)	0.000 (3)	0.024 (3)	−0.009 (3)
C8	0.041 (2)	0.041 (3)	0.036 (2)	0.0028 (19)	0.0087 (18)	0.0055 (19)
C9	0.043 (2)	0.034 (2)	0.0299 (19)	−0.0021 (18)	0.0073 (17)	0.0037 (17)
C10	0.044 (2)	0.058 (3)	0.037 (2)	0.001 (2)	0.0133 (19)	0.001 (2)
C11	0.048 (2)	0.040 (3)	0.030 (2)	0.0028 (19)	0.0099 (18)	0.0008 (17)
C12	0.030 (2)	0.049 (3)	0.041 (2)	−0.0009 (19)	0.0060 (17)	−0.004 (2)
C13	0.051 (3)	0.044 (3)	0.045 (2)	0.000 (2)	0.016 (2)	0.002 (2)
C14	0.051 (3)	0.048 (3)	0.050 (3)	0.004 (2)	0.016 (2)	−0.006 (2)
C15	0.038 (2)	0.053 (3)	0.040 (2)	0.004 (2)	0.0078 (18)	0.003 (2)
C16	0.064 (3)	0.049 (3)	0.056 (3)	−0.007 (2)	0.018 (2)	0.005 (2)
C17	0.068 (3)	0.047 (3)	0.053 (3)	−0.005 (2)	0.023 (2)	−0.008 (2)
C18	0.059 (3)	0.074 (4)	0.045 (3)	0.003 (3)	0.016 (2)	0.005 (3)
C19	0.039 (2)	0.050 (3)	0.037 (2)	0.002 (2)	0.0039 (18)	−0.003 (2)
C20	0.039 (2)	0.042 (3)	0.031 (2)	0.0013 (19)	0.0056 (17)	−0.0026 (18)
C21	0.056 (3)	0.037 (3)	0.033 (2)	0.001 (2)	0.0115 (19)	0.0004 (18)
C22	0.050 (3)	0.051 (3)	0.039 (2)	0.000 (2)	0.0152 (19)	0.003 (2)
F1	0.169 (4)	0.117 (3)	0.075 (2)	0.048 (3)	0.059 (2)	−0.008 (2)
F2	0.112 (3)	0.133 (3)	0.0448 (17)	0.011 (2)	0.0194 (17)	0.0211 (19)
F3	0.090 (3)	0.313 (7)	0.089 (3)	−0.080 (4)	0.064 (2)	−0.076 (3)
F4	0.226 (5)	0.101 (3)	0.092 (3)	0.061 (3)	0.098 (3)	0.013 (2)
F5	0.147 (4)	0.181 (4)	0.0498 (19)	0.073 (3)	0.024 (2)	0.027 (2)
F6	0.158 (4)	0.329 (8)	0.090 (3)	−0.157 (5)	0.085 (3)	−0.081 (4)
N1	0.0384 (19)	0.046 (2)	0.0310 (17)	−0.0039 (16)	0.0083 (14)	−0.0060 (15)
N2	0.038 (2)	0.062 (3)	0.0349 (18)	−0.0076 (18)	0.0106 (15)	−0.0049 (17)
N3	0.065 (3)	0.036 (2)	0.0355 (19)	0.0040 (18)	0.0157 (19)	0.0006 (16)
N4	0.0377 (19)	0.055 (2)	0.0325 (17)	−0.0058 (16)	0.0077 (14)	−0.0091 (16)
N5	0.044 (2)	0.053 (2)	0.0353 (18)	−0.0043 (18)	0.0119 (15)	−0.0030 (16)
N6	0.081 (3)	0.034 (2)	0.037 (2)	0.002 (2)	0.014 (2)	−0.0004 (17)
O1	0.0428 (17)	0.088 (3)	0.0343 (16)	0.0002 (17)	0.0013 (13)	−0.0087 (16)
O2	0.078 (2)	0.073 (3)	0.0379 (17)	−0.006 (2)	0.0045 (17)	−0.0150 (17)
O3	0.073 (2)	0.056 (2)	0.0556 (19)	0.0123 (17)	0.0336 (17)	−0.0024 (16)
O4	0.0457 (18)	0.097 (3)	0.0468 (18)	−0.0171 (18)	0.0080 (15)	−0.0243 (18)
O5	0.101 (3)	0.070 (3)	0.0410 (18)	−0.009 (2)	0.0067 (19)	−0.0184 (17)
O6	0.092 (3)	0.058 (2)	0.054 (2)	0.0106 (19)	0.0380 (19)	0.0038 (16)
S1	0.0430 (6)	0.0545 (8)	0.0309 (5)	−0.0044 (5)	0.0063 (4)	−0.0053 (5)
S2	0.0490 (7)	0.0570 (8)	0.0335 (5)	−0.0026 (6)	0.0046 (5)	−0.0070 (5)

### Geometric parameters (Å, º)

**Table:**

C1—C2	1.379 (6)	C13—C14	1.378 (6)
C1—C6	1.385 (6)	C13—H13	0.9300
C1—C8	1.483 (5)	C14—C15	1.367 (6)
C2—C3	1.378 (6)	C14—H14	0.9300
C2—H2	0.9300	C15—C16	1.379 (6)
C3—C4	1.374 (6)	C15—C18	1.482 (6)
C3—H3	0.9300	C16—C17	1.374 (6)
C4—C5	1.375 (6)	C16—H16	0.9300
C4—C7	1.488 (6)	C17—H17	0.9300
C5—C6	1.372 (6)	C18—F6	1.266 (6)
C5—H5	0.9300	C18—F5	1.283 (6)
C6—H6	0.9300	C18—F4	1.297 (6)
C7—F3	1.275 (6)	C19—O4	1.213 (5)
C7—F2	1.303 (6)	C19—N4	1.365 (5)
C7—F1	1.342 (7)	C20—N5	1.314 (5)
C8—O1	1.205 (5)	C20—N4	1.363 (5)
C8—N1	1.379 (5)	C20—S2	1.724 (4)
C9—N2	1.314 (5)	C21—C22	1.339 (6)
C9—N1	1.358 (5)	C21—N6	1.425 (5)
C9—S1	1.723 (4)	C21—S2	1.716 (4)
C10—C11	1.333 (6)	C22—N5	1.358 (5)
C10—N2	1.359 (5)	C22—H22	0.9300
C10—H10	0.9300	N1—H1	0.8600
C11—N3	1.417 (5)	N3—O3	1.220 (4)
C11—S1	1.719 (4)	N3—O2	1.227 (5)
C12—C17	1.375 (6)	N4—H4	0.8600
C12—C13	1.379 (6)	N6—O5	1.224 (5)
C12—C19	1.491 (5)	N6—O6	1.227 (5)
			
C2—C1—C6	119.5 (4)	C13—C14—H14	119.7
C2—C1—C8	122.8 (4)	C14—C15—C16	119.8 (4)
C6—C1—C8	117.7 (4)	C14—C15—C18	121.3 (4)
C3—C2—C1	120.0 (4)	C16—C15—C18	118.9 (4)
C3—C2—H2	120.0	C17—C16—C15	120.0 (4)
C1—C2—H2	120.0	C17—C16—H16	120.0
C2—C3—C4	120.1 (4)	C15—C16—H16	120.0
C2—C3—H3	120.0	C16—C17—C12	120.2 (4)
C4—C3—H3	120.0	C16—C17—H17	119.9
C5—C4—C3	120.2 (4)	C12—C17—H17	119.9
C5—C4—C7	120.0 (4)	F6—C18—F5	106.1 (5)
C3—C4—C7	119.8 (4)	F6—C18—F4	105.9 (5)
C6—C5—C4	119.9 (4)	F5—C18—F4	102.8 (5)
C6—C5—H5	120.0	F6—C18—C15	114.0 (4)
C4—C5—H5	120.0	F5—C18—C15	112.9 (4)
C5—C6—C1	120.2 (4)	F4—C18—C15	114.2 (4)
C5—C6—H6	119.9	O4—C19—N4	121.1 (4)
C1—C6—H6	119.9	O4—C19—C12	123.8 (4)
F3—C7—F2	108.3 (5)	N4—C19—C12	115.2 (3)
F3—C7—F1	106.3 (5)	N5—C20—N4	120.7 (3)
F2—C7—F1	102.9 (5)	N5—C20—S2	116.8 (3)
F3—C7—C4	114.0 (5)	N4—C20—S2	122.5 (3)
F2—C7—C4	113.4 (4)	C22—C21—N6	126.4 (4)
F1—C7—C4	111.2 (5)	C22—C21—S2	112.9 (3)
O1—C8—N1	120.6 (4)	N6—C21—S2	120.6 (3)
O1—C8—C1	123.9 (4)	C21—C22—N5	114.7 (4)
N1—C8—C1	115.6 (3)	C21—C22—H22	122.7
N2—C9—N1	120.6 (3)	N5—C22—H22	122.7
N2—C9—S1	116.6 (3)	C9—N1—C8	122.4 (3)
N1—C9—S1	122.8 (3)	C9—N1—H1	118.8
C11—C10—N2	115.0 (4)	C8—N1—H1	118.8
C11—C10—H10	122.5	C9—N2—C10	109.3 (3)
N2—C10—H10	122.5	O3—N3—O2	124.0 (4)
C10—C11—N3	126.8 (4)	O3—N3—C11	118.1 (4)
C10—C11—S1	112.6 (3)	O2—N3—C11	117.9 (4)
N3—C11—S1	120.6 (3)	C19—N4—C20	123.2 (3)
C17—C12—C13	119.9 (4)	C19—N4—H4	118.4
C17—C12—C19	118.6 (4)	C20—N4—H4	118.4
C13—C12—C19	121.6 (4)	C20—N5—C22	109.4 (3)
C14—C13—C12	119.6 (4)	O5—N6—O6	124.0 (4)
C14—C13—H13	120.2	O5—N6—C21	117.8 (4)
C12—C13—H13	120.2	O6—N6—C21	118.2 (4)
C15—C14—C13	120.6 (4)	C11—S1—C9	86.47 (19)
C15—C14—H14	119.7	C21—S2—C20	86.26 (19)
			
C6—C1—C2—C3	−0.6 (6)	C16—C15—C18—F4	166.3 (5)
C8—C1—C2—C3	−179.4 (4)	C17—C12—C19—O4	−50.0 (6)
C1—C2—C3—C4	−0.6 (7)	C13—C12—C19—O4	129.2 (5)
C2—C3—C4—C5	0.7 (7)	C17—C12—C19—N4	129.0 (4)
C2—C3—C4—C7	−177.9 (4)	C13—C12—C19—N4	−51.8 (6)
C3—C4—C5—C6	0.4 (7)	N6—C21—C22—N5	177.4 (4)
C7—C4—C5—C6	179.1 (5)	S2—C21—C22—N5	0.2 (5)
C4—C5—C6—C1	−1.7 (7)	N2—C9—N1—C8	175.9 (4)
C2—C1—C6—C5	1.8 (7)	S1—C9—N1—C8	−2.5 (6)
C8—C1—C6—C5	−179.4 (4)	O1—C8—N1—C9	4.8 (6)
C5—C4—C7—F3	17.1 (8)	C1—C8—N1—C9	−173.6 (4)
C3—C4—C7—F3	−164.2 (5)	N1—C9—N2—C10	−177.1 (4)
C5—C4—C7—F2	−107.4 (6)	S1—C9—N2—C10	1.3 (5)
C3—C4—C7—F2	71.3 (7)	C11—C10—N2—C9	−0.6 (6)
C5—C4—C7—F1	137.3 (5)	C10—C11—N3—O3	2.6 (7)
C3—C4—C7—F1	−44.1 (7)	S1—C11—N3—O3	−178.0 (3)
C2—C1—C8—O1	149.3 (4)	C10—C11—N3—O2	−177.3 (4)
C6—C1—C8—O1	−29.5 (6)	S1—C11—N3—O2	2.1 (5)
C2—C1—C8—N1	−32.4 (6)	O4—C19—N4—C20	2.8 (7)
C6—C1—C8—N1	148.8 (4)	C12—C19—N4—C20	−176.2 (4)
N2—C10—C11—N3	179.1 (4)	N5—C20—N4—C19	170.2 (4)
N2—C10—C11—S1	−0.3 (5)	S2—C20—N4—C19	−9.0 (6)
C17—C12—C13—C14	0.0 (7)	N4—C20—N5—C22	−178.9 (4)
C19—C12—C13—C14	−179.2 (4)	S2—C20—N5—C22	0.4 (5)
C12—C13—C14—C15	−1.2 (7)	C21—C22—N5—C20	−0.3 (6)
C13—C14—C15—C16	0.8 (7)	C22—C21—N6—O5	172.6 (4)
C13—C14—C15—C18	−177.6 (4)	S2—C21—N6—O5	−10.3 (6)
C14—C15—C16—C17	0.8 (7)	C22—C21—N6—O6	−7.9 (7)
C18—C15—C16—C17	179.2 (4)	S2—C21—N6—O6	169.2 (3)
C15—C16—C17—C12	−2.0 (7)	C10—C11—S1—C9	0.8 (4)
C13—C12—C17—C16	1.6 (7)	N3—C11—S1—C9	−178.7 (4)
C19—C12—C17—C16	−179.2 (4)	N2—C9—S1—C11	−1.2 (3)
C14—C15—C18—F6	−137.1 (6)	N1—C9—S1—C11	177.2 (4)
C16—C15—C18—F6	44.5 (7)	C22—C21—S2—C20	0.0 (4)
C14—C15—C18—F5	101.7 (6)	N6—C21—S2—C20	−177.4 (4)
C16—C15—C18—F5	−76.7 (6)	N5—C20—S2—C21	−0.3 (4)
C14—C15—C18—F4	−15.3 (7)	N4—C20—S2—C21	179.0 (4)

### Hydrogen-bond geometry (Å, º)

**Table:**

*D*—H···*A*	*D*—H	H···*A*	*D*···*A*	*D*—H···*A*
N1—H1···N5^i^	0.86	2.32	3.044 (5)	142
N1—H1···O3^i^	0.86	2.57	3.023 (5)	115
N4—H4···N2^ii^	0.86	2.18	2.939 (5)	147
N4—H4···O2	0.86	2.62	3.146 (5)	121

Symmetry codes: (i) −*x*, *y*−1/2, −*z*+1/2; (ii) −*x*, *y*+1/2, −*z*+1/2.
